# Development and validation of a novel detection method for *Rickettsia rickettsii* using a loop-mediated isothermal amplification assay

**DOI:** 10.3389/fmicb.2023.1276809

**Published:** 2024-01-08

**Authors:** Bertha I. Carvajal-Gamez, Aída Olguín-Barrera, Luis Tinoco-Gracia, Guadalupe Gordillo-Perez, Karla Dzul-Rosado, Gabriela Aguilar-Tipacamú, Mario Hidalgo-Ruiz, Juan Mosqueda

**Affiliations:** ^1^Immunology and Vaccines Laboratory, Natural Sciences College, Autonomous University of Queretaro, Queretaro, Mexico; ^2^Cuerpo Academico, Salud Animal y Microbiologia Ambiental, Natural Sciences College, Autonomous University of Queretaro, Queretaro, Mexico; ^3^Maestria en Salud y Producción Animal Sustentable, Facultad de Ciencias Naturales, Universidad Autónoma de Querétaro, Querétaro, Mexico; ^4^Laboratorio de Salud Pública Veterinaria, Instituto de Investigaciones en Ciencias Veterinarias, Universidad Autónoma de Baja California, Baja California, Mexico; ^5^Unidad de Investigación Médica en Enfermedades Infecciosas, Hospital de Pediatría, Centro Medico Nacional SXXI-Instituto Mexicano del Seguro Social, Mexico City, Mexico; ^6^Dr. Hideyo Noguchi Regional Research Center, Autonomous University of Yucatan, Mérida, Mexico

**Keywords:** Rocky Mountain spotted fever, diagnostics, tick-borne pathogens, isothermal amplification, *Rickettsia*

## Abstract

**Introduction:**

*Rickettsia rickettsii* is an obligate, intracellular pathogen and the causative agent of Rocky Mountain spotted fever (RMSF). RMSF is an important zoonotic disease due to its high fatal outcome in humans. The difficulty of clinical diagnosis due to the low sensitivity and specificity of current diagnostic methods are a principal setback. We reported the development of a new method for the detection of *R. rickettsii* in human and tick DNA samples using loop-mediated isothermal amplification (LAMP), as well as the validation of the LAMP test for *R. rickettsii* in field samples of infected ticks and humans, determining the diagnostic sensitivity and specificity, as well as the reproducibility of the test.

**Methods:**

This technique uses hydroxy naphthol blue (HNB) as an indicator of the formation of magnesium pyrophosphate, a marker for the presence of DNA. Here, we used a putative *R. rickettsii* gene as a target for three pairs of primers that specifically amplify *R. rickettsii* DNA by hairpin-based isothermal amplification technique (LAMP).

**Results and discussion:**

The sensitivity of the assay was ~1.6–3 pg, which is 10 times more sensitive than PCR. To determine the diagnostics specificity and sensitivity, 103 human DNA samples and 30 tick DNA samples were evaluated. For the human samples, a sensitivity for HNB of 93%, a specificity of 70% and a k of 0.53 were obtained. For electrophoresis the sensitivity was 97% with a specificity of 58% and a k of 0.42. For tick samples, a sensitivity of 80% was obtained, a specificity of 93% for HNB and for electrophoresis the sensitivity and specificity were 87%. The k for both was 0.73. The degree of concordance between HNB and electrophoresis was 0.82 for humans and for ticks, it was 0.87. The result is obtained in shorter time, compared to a PCR protocol, and is visually interpreted by the color change. Therefore, this method could be a reliable tool for the early diagnosis of rickettsiosis.

## 1 Introduction

*Rickettsia rickettsii* is responsible for Rocky Mountain spotted fever (RMSF) and the most important vectors are Ixodid ticks, including *Rhipicephalus sanguineus* (tropical lineage, now classified as *R. linnaei*)*, Dermacentor andersoni, D. variabilis, Amblyomma sculptum, A. americanum*, and *A. patinoi* (Pinter et al., 2011; Piranda et al., 2011; Dergousoff et al., 2013; Parola et al., 2013; Levin et al., 2017; Costa et al., 2020; Martínez-Diaz et al., 2021; Šlapeta et al., 2022; Sánchez Pérez et al., 2023). RMSF occurs after the bite of an infected tick, with an incubation period of 7 days. The signs and symptoms vary in severity with elevated body temperature (38–40°C) and a rash that initially appears on the wrists and ankles, gradually spreading over time. RMSF is characterized by proinflammatory and procoagulant changes, the development of systemic vasculitis, and vascular damage affecting various organs, including the brain, pancreas, lungs, liver, skeletal muscles, kidneys, and skin. Such multisystemic injuries lead to increased vascular permeability, edema, hypovolemia, and hypotension (Helmick et al., 1984; Weber and Walker, 1991; Ibarra Stone et al., 2022).

There are manifold complications of rickettsiosis, including pneumonia, myocardial edema, portal triaditis and vasculitis, thrombocytopenia or severe coagulopathies, hypercoagulable state, encephalomyelitis and meningoencephalitis, gangrene or cutaneous necrosis, acute renal failure, central nervous system abnormalities, coma as a neurological sequela, and death (Walker et al., 1980a,b; Randall and Walker, 1984; Roggli et al., 1985; Jackson et al., 1986; Horney and Walker, 1988; Hove and Walker, 1995; Conlon et al., 1996; Sundy et al., 1996; Bergeron et al., 1997; Wei and Baumann, 1999). To prevent these complications, a rapid and specific diagnostic method is required. The diagnosis of rickettsiosis is complex due to the expansive clinical manifestations, often mistaken for symptoms of dengue or chikungunya (Álvarez-López et al., 2021). Furthermore, since symptoms typically manifest within the first 2 weeks post-infection, outcomes can be fatal in the absence of early therapeutic intervention. For this reason, treatment must be administered as early as possible—even if the infection has not yet been confirmed (Kirkland et al., 1995). Hence, there is a need for tests that enable swift and simple interpretation. When coupled with clinical and paraclinical characteristics, these tests allow a better approach to patient care and enhance the prognosis for survival.

Methods for specific diagnosis are based on molecular and serological tests. Methods based on PCR amplification of gene fragments, such as htrA, 16S ribosomal RNA, gltA and ompB, among others (Oteo et al., 2014), have been extensively explored for their diagnostic utility. All of these are highly conserved in species of *Rickettsia* and have been used to develop diagnostic methods based on semi-quantitative and quantitative PCR and real-time multiplex PCR to detect Rickettsia DNA presence. Some of these techniques are laborious and time-consuming (Roux and Raoult, 1993, 1995; Roux et al., 1996; Eremeeva et al., 2003; Tomita et al., 2008; Nakao et al., 2013; Ueno et al., 2016).

The indirect fluorescent antibody (IFA) test stands as the most commonly used serological diagnostic test for detecting the presence of specific antibodies; however, this technique cannot detect the presence of *Rickettsia* in an acute state because its respective antibodies appear only 10–14 days after infection (Paddock et al., 2002). Serological techniques exhibited limited specificity as IgM and IgG antibodies cannot distinguish between infections caused by *R. rickettsii* and those induced by other pathogens that cause spotted fever, such as *R. akari* and *R. conorii* (Kaplan and Schonberger, 1986; Sexton and Kaye, 2002).

The hairpin-based isothermal amplification method (LAMP) has proven to be the most efficient, economical, and specific molecular diagnostic method for the diagnosis of murine typhus and scrub typhus (Dittrich et al., 2014; Roy et al., 2021). It uses a constant amplification temperature and six primers that recognize six unique sites in the DNA sequence (Notomi et al., 2000).

To date, there are no reports of a test to detect the specific species of *R. rickettsii*. This study aims to develop a LAMP test for the detection of *R. rickettsii* DNA in human and tick samples, as well as to evaluate the sensitivity, specificity, and reproducibility of the test.

## 2 Materials and methods

### 2.1 Controls DNA samples

*R. rickettsii* DNA positive control of human blood was obtained by Luis Tinoco-Gracia (Autonomous University of Baja California). DNA from *R. typhi* (PMID: 25076014), *R. japonica, R. helvetica, R. australis, R. conorii, R. felis, R. prowasekii, R. africae, R. parkeri* (strain Atlantic Rainforest), and *R. slovaca* were obtained by Karla Dzul Rosado and Cesar Lugo (Autonomous University of Yucatan), and which were then analyzed and sequenced, under the collaboration of the UTMB. Dr. Jorge Zavala Castro. Marisa Farber (National Agricultural Technology Institute, Argentina) obtained the DNA of *Borrelia burgdorferi* and *Anaplasma phagocytophilum*. The DNA of the *R. rickettsii* reference strain was extracted from a vial of *R. rickettsii* containing yolk sac antigen (RA2296, *R. rickettsii* IFA antigen, Sheila Smith strain, Biologics Branch Scientific Resources Program, CDC, Atlanta, GA) using the illustra™ blood genomicPrep Mini Spin Kit (GE Healthcare, Chigago, IL, USA), according to the manufacturer's instructions. All DNA control samples used in this study were previously confirmed by sequencing. The negative control for each assay consists of the addition of nuclease-free water.

### 2.2 Clinical samples

The Infectious Diseases Research Unit of the Centro Médico Nacional Siglo XXI in Mexico City (UIMEIP-CMNSXXI) provided 77 DNA samples from patients, while the Instituto de Investigaciones en Ciencias Veterinarias de la Autonomous University of Baja California (IICV-UABC) contributed 26 samples. A total of 30 tick DNA samples were also donated by the same institution, with the ticks being brown dog ticks collected from dogs in RMSF endemic areas. In total, there were 133 human and tick DNA samples. The samples used for diagnostic sensitivity, specificity, and reproducibility were obtained from patients suspected of Rickettsial disease. These blood samples were taken from a population in RMSF-endemic areas in the Northwest and Southeast of the country during the spring-summer period. Negative controls were obtained from humans in non-endemic areas of RMSF and from patients with undifferentiated febrile illness. All samples were previously determined as positive or negative using a previously published PCR protocol for the presence of *R. rickettsii* DNA (Eremeeva et al., 2003), along with sequencing and clinical disease assessment (Mexican Official STANDARD NOM-032-SSA2-2014, 2015; Tinoco-Gracia et al., 2018).

### 2.3 Design of species-specific primers for the *R. rickettsii* LAMP

For this study, two *R. rickettsii* genome sequences were obtained from GenBank: the Iowa Strain (accession number: CP000766) and the Sheila Smith reference strain (accession number: CP000766). A nucleotide sequence of *R. rickettsii* coding for a putative gene of unknown function (AF042063.1) was subjected to a BLAST analysis (http://blast.ncbi.nlm.nih.gov/Blast.cgi), and the nucleotide sequences of this gene from different strains of *R. rickettsii* (Colombia, Brazil, Sheila Smith, Morgan, Iowa, Hauke, Hino, Arizona, Hlp#2) were aligned and compared in MUSCLE program (http://www.ebi.ac.uk/Tools/msa/muscle/) to identify conserved regions.

A set of species-specific primers complementary to the *R. rickettsii* gene sequences of each isolate was designed according to the recommendations by Notomi et al. (2000). The designed primers were analyzed using BLAST (https://blast.ncbi.nlm.nih.gov/Blast.cgi) and verified for specificity to *R. rickettsia*. We considered all *Rickettsia* records reported for human and/or animal hosts. A set of six primers was generated and there is an ongoing patent application for this set (No. MX/a/2022/003615). The alignment includes the following *Rickettsia* species: *R. amblyommatis, R. montanensis, R. rhipicephali, R. massiliae, R. japonica, R. heilongjiangensis, R. africae, R. philipii, R. slovaca, R. parkeri* (strain Atlantic Rainforest), *R. felis, R. akari, R. peacockii, R. bellii, R. australis, R. peacockii, R. conorii, R. rhipicephali*, and *R. massiliae*.

### 2.4 Cloning and sequencing

Cloning was carried out in the pCR™ 4-TOPO^®^ TA cloning maintenance vector (Invitrogen, Waltham, MA, USA) following the manufacturer's instructions. Subsequently, DH5α cells (*E. coli* TOP 10) were transformed, and the transformed cells were plated in petri dish with LB-ampicillin medium (100 mg/ml), incubated at 37°C for 18 h. As a control, the transformation was carried out without the PCR product. Furthermore, ten bacteria colonies were selected and used for the isolation and purification of the plasmids containing the insert and were prepared for commercial sequencing. The cloning products were sequenced in both directions using each of the primers by the automated Sanger method.

### 2.5 PCR assay using specific primers for *R. rickettsii* detection

A previously published PCR protocol was used as a comparative assay. Briefly, PCR amplification was carried out using the RR190.547F primer (5′-CCT GCC GAT AAT TAT ACA GGT TTA-3′) and RR190.701R primer (5′-GTT CCG TTA ATG GCA GCA T-3′) (Eremeeva et al., 2003). Then, the protocol consisted of an initial denaturation at 94°C for 1 min, 34 cycles at 94°C for 1 min, followed by an annealing step at 63°C for 45 s and an extension step of 71°C for 45 s. The final extension was carried out at 72°C for 7 min. Each primer was used as a concentration of 10 pmol using MyTaq™ mix (Bioline, London, UK). An amplicon of ~154 bp was obtained, and PCR was performed in a final volume of 25 μL containing a mixture of 30 ng of total *R. rickettsii* DNA. The PCR amplification products were analyzed by electrophoresis on a 1.5% agarose gel and stained with GelRed^®^ (Biotium, Hayward, CA, USA).

### 2.6 LAMP protocol

The protocol for LAMP was developed following the method described by Notomi et al. (2000). Briefly, the reaction was performed in a final volume of 25 μL of a mixture containing 30 ng of the DNA sample, 4 μM each of the FIPRrickettsii and BIPRrickettsii primers, 0.2 μM each of the F3Rrickettsii, B3Rrickettsii, LPRrickettsii, and LFRrickettsii primers, 6 mM MgSO_4_ (New England Biolabs, Hitchin, UK), 0.8 M betaine (Thermo Fisher Scientific, Waltham, MA, USA), 1.4 mM of dNTPs (Invitrogen, Carlsbad, CA, USA), 20 μM of hydroxy naphthol blue (HNB; Sigma-Aldrich, USA), LAMP buffer (20 mM Tris-HCl, pH 8.8), and 8 U of Bst DNA polymerase (New England Biolabs, Hitchin, UK). The temperature of the amplification was evaluated by exposing the reaction to 61, 63, or 65°C for 60 min followed by 80°C for 3 min. The LAMP products were analyzed by electrophoresis on a 1.5% agarose gel and visualized under an ultraviolet (UV) light after staining with GelRed^®^ (Biotium, Hayward, CA, USA). Each LAMP reaction was performed three times.

### 2.7 Standardization and optimization of the LAMP assay

The LAMP assay for analytical sensitivity determination was evaluated using 10-fold serial dilutions of *R. rickettssi* DNA. The initial concentration of *R. rickettsii* DNA was 30 ng. The same DNA sample was also evaluated by PCR, which was used as a control, as described above. The analytical specificity of the LAMP assay for *R. rickettsii* was evaluated using DNA from different species, such as *R. typhi, R. japonica, R. helvetica, R. australis, R. conorii, R. felis, R. prowasekii, R. africae, R. parkeri* (strain Atlantic Rainforest), *R. slovaca, Ehrlichia canis, Borrelia burgdorferi*, and *Anaplasma phagocytophilum*. The assay was also tested using the DNA samples from human, dog (*Canis lupus familiaris*), and the brown dog tick from Mexicali, BC, Mexico (Almazán et al., 2023). In each evaluation, the LAMP reaction was performed at 63°C for 60 min and compared with the results of the PCR assay. To evaluate the incubation duration of the test, the LAMP reaction was incubated for different durations: 30, 45, and 60 min at 63°C, then at 80°C for 3 min. The result of each assay was visualized on a 1.5% agarose gel stained with GelRed^®^ (Biotium, Hayward, CA, USA). All assays were performed three times at different times.

To validate the assay, we followed two referenced protocols for diagnostics tests (Banoo, 2010; OIE, 2018). Therefore, the following methodology was performed according to both manuals.

### 2.8 Analytical reproducibility test of the LAMP method

The reproducibility test was performed using two different strains of *R. rickettsii* DNA: DNA from the *R. rickettsii* reference strain obtained from yolk sac and DNA from *R. rickettsii* obtained from a clinical sample previously confirmed by sequencing. In addition, a previously analyzed and sequenced plasmid DNA construct was used in the evaluation. All standardization, optimization, sensitivity, and specificity tests were performed three times.

### 2.9 Blind study for the evaluation of samples with the LAMP method

To assess the suitability of the LAMP technique, 103 human DNA samples and 30 tick DNA samples were evaluated, following the protocol, using positive and negative controls, in a blind study. The result of the test was determined in agarose gel stained with red gel and visualized in the digital imaging system (Bio-Rad Gel Doc Imager, Wayne, PA, USA). Additionally, the results were evaluated visually, by changing the color from purple to blue. To determine the repeatability of the technique, six random samples were taken, previously determined as positive in the LAMP technique. The following day, the same protocol was repeated, using the same lot of reagents and equipment. The obtained results were then compared. In addition, data such as availability of equipment, interpretation capacity, test performance, time necessary for the delivery of results, and the feasibility of the test to be carried out in their laboratories were obtained. The LAMP method was performed three times.

### 2.10 Determination of specificity and diagnostic sensitivity

The results obtained were evaluated in a 2 × 2 contingency table to obtain the specificity and sensitivity values of the LAMP method. The sensitivity is calculated as [true positive/(true positive + false negative)] and the specificity as [true negative/(true negative + false positive)]. Both positive and negative predictive values, the degree of concordance using the Kappa index (k) (Landis and Koch, 1977), the likelihood ratios, as well as the precision of the new method were also estimated (Diagnostic Test Calculator http://araw.mede.uic.edu/cgi-bin/testcalc.pl; MEDCALC easy-to-use statistical software https://www.medcalc.org/index.php). The determination of specificity and diagnostic sensitivity was performed three times at various intervals.

## 3 Results

### 3.1 Design of *R. rickettsii*-specific primers

**Figure 1:**
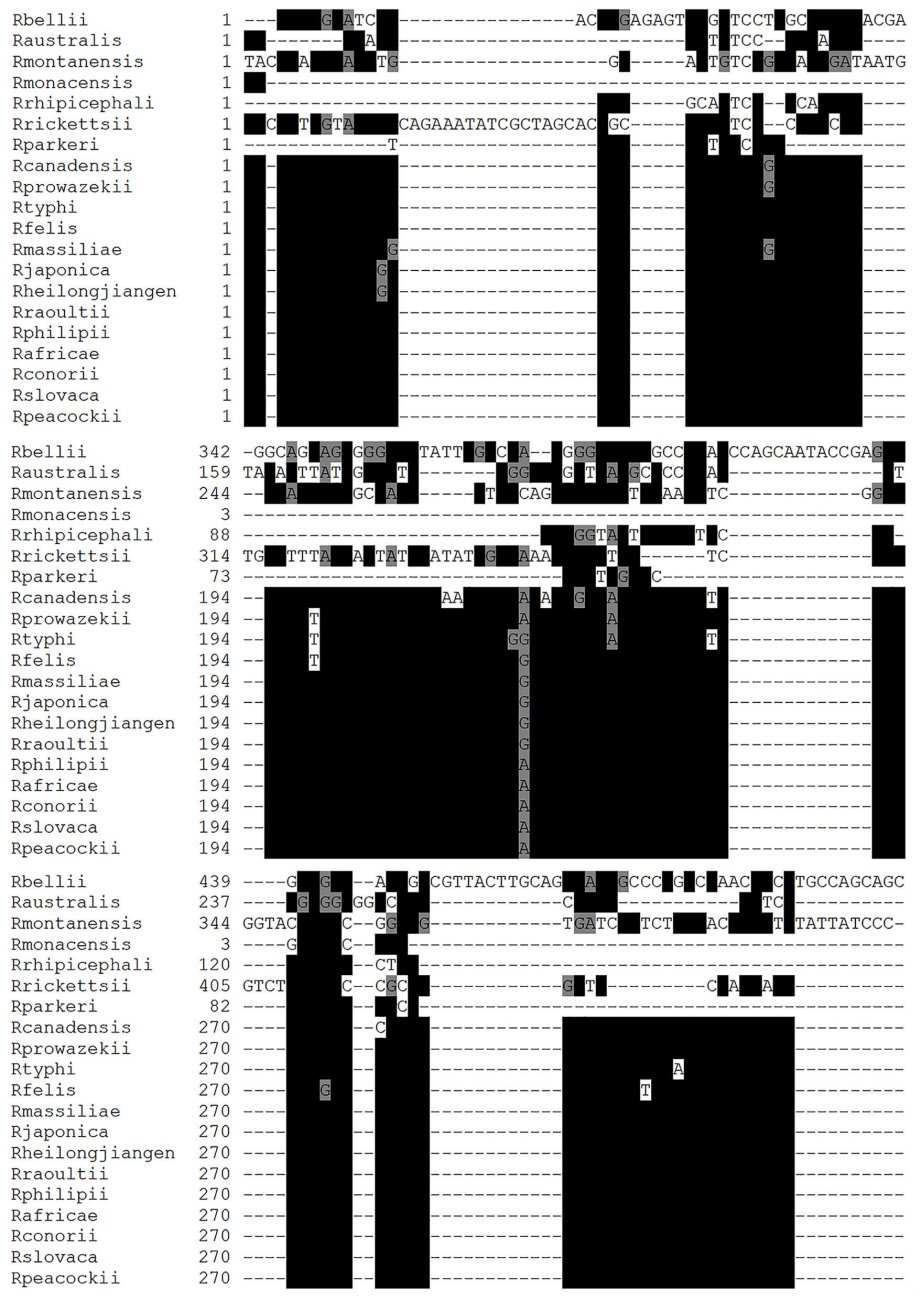
Multiple alignment using the nucleic acid sequences of *Rickettsia* species. Nucleic acid sequences of unknown genes from *Rickettsia rickettsii* (*R. rickettsii*), *Rickettsia amblyommii* (*R. amblyommatis*), *Rickettsia montamensis* (*R. montamensis*), *Rickettsia rhipicephali* (*R. rhipicephali*), *Rickettsia massiliae* (*R. massiliae*), *Rickettsia japonica* (*R. japonica*), *Rickettsia heilongjiangensis* (*R. heilongjiangensis*), *Rickettsia africae* (*R. africae*), *Rickettsia philipii* (*R. philipii*), *Rickettsia slovaca* (*R. slovaca*), *Rickettsia parkeri* (*R. parkeri*), *Rikettsia felis* (*R. felis*), *Rickettsia akari* (*R. akari*), *Rickettsia peacockii* (*R. peacockii*), *Rickettsia bellil* (*R. bellil*), *Rickettsia australis* (*R. australis*), *Rickettsia conorii* (*R. conorii*), and *Rickettsia rhipicephali* (*R. rhipicephali*). The alignments were generated using the MUSCLE program and edited by the BOXSHADE program. The black box shows homologous sequences indicated with *, and the gray box denotes similar sequences.

The LAMP primers designed for *R. rickettsii* are shown in Table 1. They were designed based on the unknown gene sequence of *R. rickettsia* (AF042063.1), which was used for the design of primers in the conserved and specific regions of this sequence in *R. rickettsii* isolates (Figure 1). Multiple alignments of the unknown gene DNA sequences showed a high degree of conservation between the different *R. rickettsii* isolates used in this study as well as a high degree of divergence with other species of *Rickettsia*, except *R. phillipi* (Figure 1). Comparative analysis of the unknown gene sequences between *R. rickettsii* and *R. phillipi* showed an enzymatic digestion site with the *Psi*I enzyme that distinguishes between the sequences in these two species (Table 1).

**Table 1:** >AF042063.1 *Rickettsia rickettsii* putative protease IV (cjsT) gene, complete cds; and unknown genes.

ACTAAACACTCATCTGATAACGAATTGGTGTTTATTAAGAGTTAAAATAGTGGAGGTGAAATACCAATAG	
AAATACGTCAAGTTATTAACTTTAATTAAAAAACTTTATAATACATGATGCCTATTTATTAATAAGTATT	
TATTATGATAAAAAATTCTAAATCTTCAGCAGTCTCAAAATTTAATCCTATAGCATTGTCATACAATAAA	
ATAATAGAGTTTAAATCACAAAAGGCTTTAATTGTAGCGGGGGAATGAATAGAACCATTAAAAAATGTAA	
AAAGTTCTATAGATGCTAGTGCAGAAATAGCAACAACTCTTTTAAACTCTTGAGATAGCATGGTGAGCTG	
GAGCATAGCCGTATACTTGACCTTCTCCTTTAATCCACAGCGTAACAAAAAACGGAAAATATGAATATCC	
TGATTGACTGATAATTTTTTGTTCAGATAAGTAGATATATTCTGAGCTATAGGCAAGTGTTGTCATTAAC	
CCTTTTCCTTTTCCACCTTTACCTCTCTGATTCTCACTTTGTGGGCTAACTATGTGCAATATCTTTACTG	
TTTCATCAGGATTCTTAGCTAGTCTTTCCTTGAAATCGGCAAAGTCTGGCCATTTAGCTGATGCAGCTTT	
AATTGGCATGC	
**Primer**	**Sequence 5'-3'**
F3 Forward-outer primer	GGC TTT AAT TGT AGC GGG GGA ATG AAT AGA
B3 Backward-outer primer	GCA TCG CAA TTA AAG CTG CAT CAG CTA A
FIP Forward-inner primer	CGC TGT GGA TTA AAG GAG AAG GTC AAG tttt CTA TAG ATG CTA GTG CAG AAA TAG CAA CAA CTC
(F1ttttF2)	
BIP Backward-inner primer	GGC AAG TGT TGT CAT TAA CCC TTT TCC TTT TCC tttt GGC CAG ACT TTG CCG ATT TCA AGG
(B1ttttB2c)	
LoopF	CGG CTA TGC TCC AGC TCA CCA T
LoopB	CCT TTA CCT CTC CTG ATT CTC ACT TTG TGG GC

### 3.2 Analytical sensitivity of the LAMP reaction

**Figure 2:**
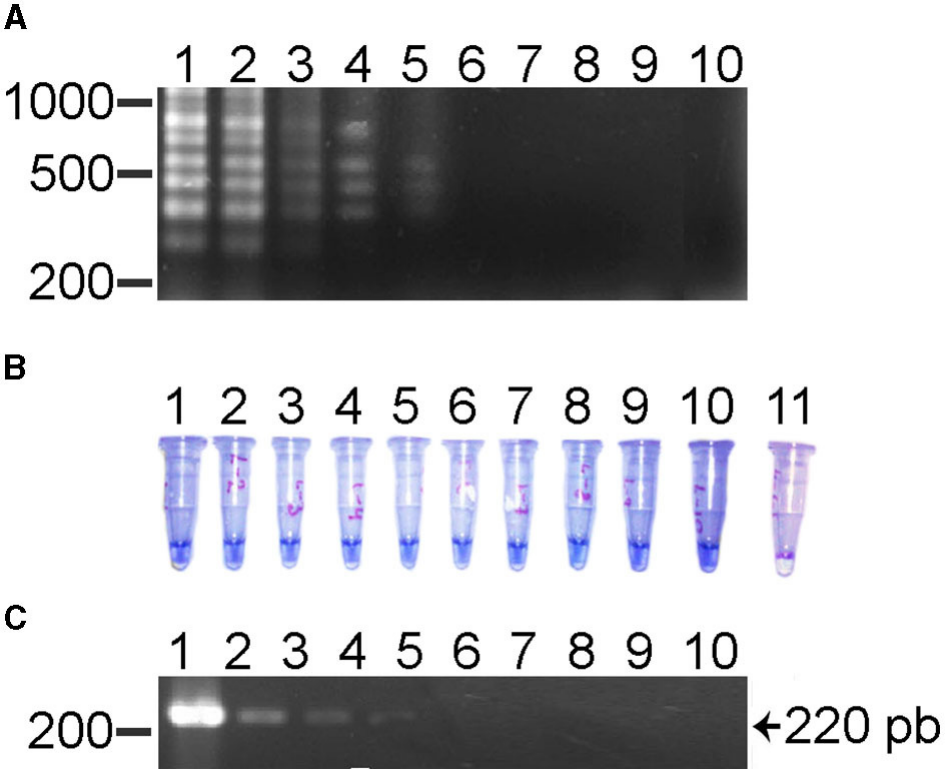
Sensitivity test for the LAMP test and its comparison with the PCR for the detection of *R. rickettsii*. **(A)** LAMP assay using serial dilutions (10^10^) with DNA of a clinical sample. Lane 1, 10^1^ or 30 ng; lane 2, 10^−2^ or 3 ng; lane 3, 10^−3^ or 0.3 ng; lane 4, 10^−4^ or 0.03 ng; lane 5, 10^−5^ or 0.003 ng; lane 6, 10^−6^ or 0.0003 ng; lane 7, 10^−7^ or 0.00003 ng; lane 8, 10^−8^ or 0.000003; lane 9, 10^−9^ or 0.0000003 ng; lane 10 is a negative control. **(B)** HNB LAMP assay using serial dilutions (10^10^) using DNA of a clinical sample. Lane 1, 10^1^ or 30 ng; lane 2, 10^−2^ or 3 ng; lane 3, 10^−3^ or 0.3 ng; lane 4, 10^−4^ or 0.03 ng; lane 5, 10^−5^ or 0.003 ng; lane 6, 10^−6^ or 0.0003 ng; lane 7, 10^−7^ or 0.00003 ng; lane 8, 10^−8^ or 0.000003; lane 9, 10^−9^ or 0.0000003 ng; lane 10 is a negative control. **(C)** PCR assay's serial dilution (10^10^) using a clinical sample DNA. Lane 1, 10^1^ or 30 ng; lane 2, 10^−2^ or 3 ng; lane 3, 10^−3^ or 0.3 ng; lane 4, 10^−4^ or 0.03 ng; lane 5, 10^−5^ or 0.003 ng; lane 6, 10^−6^ or 0.0003 ng; lane 7, 10^−7^ or 0.00003 ng; lane 8, 10^−8^ or 0.000003; lane 9, 10^−9^ or 0.0000003 ng; lane 10 is a negative control.

The sensitivity of the reaction showed a lower limit of detection for this LAMP assay was 3.0 × 10^−5^ or 0.003 ng (Figure 2). When the sensitivity was determined using a clinical positive sample, the lowest limit of detection was 1.6 × 10^−5^ or 1.6 pg. The limit of detection by conventional PCR was of 3.0 × 10^−2^ or 0.03 pg using the recombinant plasmid as positive control (Figure 2). These results show that the LAMP assay is 1,000 times more sensitive than conventional PCR for the detection of *R. rickettsii* DNA. The reproducibility of the LAMP assay was evaluated using DNA from clinical samples and analyzed three times.

### 3.3 Analytical specificity of the *R. rickettsii* LAMP assay

**Figure 3:**
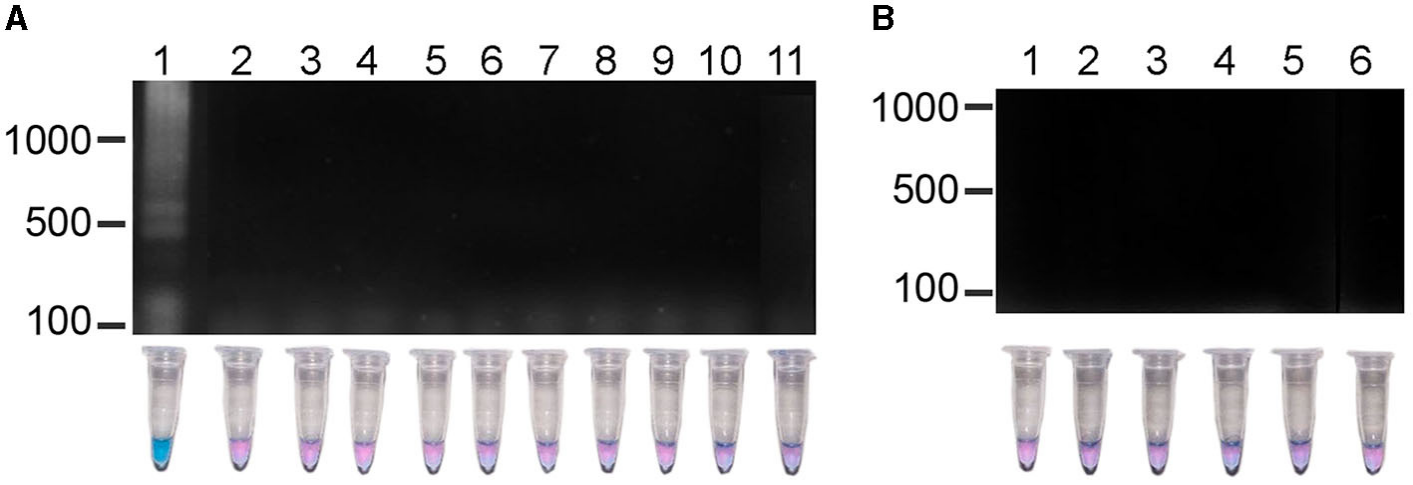
Specificity of the LAMP method in the detection *R. rickettsii*. **(A)** Lane 1, *R. rickettsii*, DNA of clinical sample; lane 2, *R. typhi*; lane 3, *R. japonica*; lane 4, *R. helvetica*; lane 5, *R. australis*; lane 6, *R. conorii*; lane 7, *R. felis*; lane 8, *R. prowasekii*; lane 9, *R. africae*; lane 10, *R. parkeri*; lane 11, *R. slovaca*. **(B)** Lane 1, *Ehrlichia chaffensiis*; lane 2, *E. cannis*; lane 3, *Borrelia burgdorferi*; lane 4, *Anaplasma phagocytophilum;* lane 5; *Anaplasma marginale*; lane 6, human DNA.

The results of experiments to evaluate the specificity of the LAMP test showed that a positive reaction was only observed in the *R. rickettsii* DNA sample; the DNA samples from other nine species of *Rickettsia* (Figure 3A) nor the *Ehrlichia, Borrelia, Anaplasma* species or the human DNA sample did not show amplification (Figure 3B). These results confirmed that the sequences of the primers aligned only with the *R. rickettsii* template, and that no false positive or non-specific amplifications were observed when other related DNA templates were used, which indicates the high specificity of this test.

### 3.4 Standardization of the reaction with HNB

**Figure 4:**
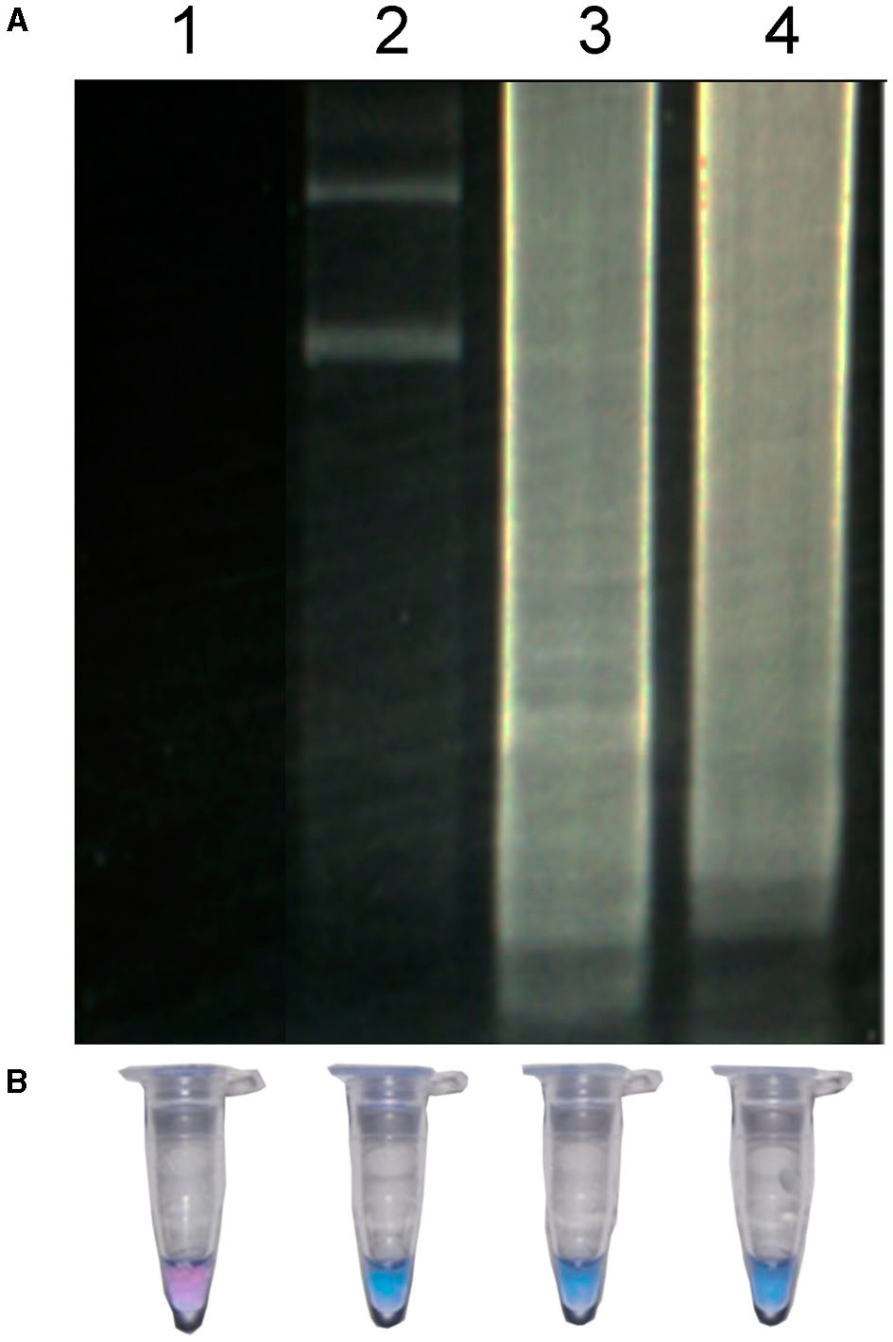
LAMP of *R. rickettsia* DNA at different times detected by agarose gel **(A)** and by visual detection **(B)**. Lane 1, negative control; lane 2, LAMP reaction for 30 min; lane 3, LAMP reaction for 45 min; LAMP reaction for 60 min.

Additionally, the minimum time necessary for DNA amplification was evaluated using the LAMP technique. As seen in Figure 4, in a reaction at 63°C, the minimum time required to observe a positive amplification on electrophoresis was 30 min (Figure 4, lane 2). The characteristic smear of a LAMP assay was observed within the 45 min and 60 min (Figure 4, lanes 3 and 4). When the use of HNB as an indicator of a positive signal was analyzed, the color of the test reaction was observed to change according to the concentration of Mg^2+^ ions. The color change of HNB in positive samples turned from violet to sky blue, in the 30 min time evaluated and did not change after this time (Figure 4, lanes 2–4), indicating a positive reaction. On the other hand, no change in color was observed in the negative control (Figure 4, lane 1).

### 3.5 Level of agreement between the PCR and LAMP technique

The results showed that in human clinical samples for LAMP-HNB, 28 samples were positive and 51 were negatives with an accuracy of 77% (95% CI: 67.3–84.5%) and *k* = 0.53 (95% CI: 0.35–0.70%); with 22 false positives and 2 false negatives (Table 2). For LAMP visualized by electrophoresis, we observed an accuracy of 69% (95% CI: 59–77.7%) and *k* = 0.42 (95% CI: 0.26–0.58%), with 29 positive samples, 42 negative samples, 31 false positive samples and one false negative. On the other hand, in tick DNA samples evaluated using the LAMP-HNB test, as shown in Table 2, 12 samples were positive, 14 were negative, one false positive and three false negative. Regarding LAMP analyzed by electrophoresis, there were 13 positive and 13 negative samples, two false positives and two false negatives. The accuracy value was 86.7% (95% CI: 69.3–96.2%) for both, the HNB and the electrophoresis methods. The *k* was 0.73 (95% CI: 0.38–1%) for both methods as well.

**Table 2:** Results using the LAMP and PCR methods in human and tick samples and their level of agreement.

	**PCR**			**Accuracy (%)**	**CI 95%**	**kappa (k)**	**CI 95%**
	**Positive**	**Negative**	**Total**				
**HUMANS**							
**LAMP-HNB**							
Positive	28	22	50	77	67.3–84.5	0.53	0.35–0.70
Negative	2	51	53				
Total	30	73	103				
**LAMP- electrophoresis**							
Positive	29	31	60	69	59–77.7	0.42	0.26–0.58
Negative	1	42	43				
Total	30	73	103				
**TICKS**							
**LAMP-HNB**							
Positive	12	1	13	86.7	69.3 96.2	0.73	0.38–1
Negative	3	14	17				
Total	15	15	30				
**LAMP- electrophoresis**							
Positive	13	2	15	86.7	69.3 96.2	0.73	0.38–1
Negative	2	13	15				
Total	15	15	30				

95% CI, 95% confidence interval.

Additionally, the concordance between colorimetry and electrophoresis was estimated as visualization methods of the LAMP test results for both human and tick samples (Table 3). For human clinical samples, out of 50 positive samples determined by electrophoresis, 50 were positive using HNB, while out of 53 negative samples determined by electrophoresis, 43 were negative using HNB, which obtained a k value of 0.82 (95% CI: 0.63–1.0%). For tick DNA samples, out of 13 positive samples determined by electrophoresis, 13 were positive when HNB was used, while out of 17 negative samples determined by electrophoresis, 15 were negative using HNB, which gave a k value of 0.87 (95% CI: 0.51–1.0%) (Table 3). This results, indicate that there is a very good agreement between both visualization methods (Landis and Koch, 1977).

**Table 3:** Results using the LAMP-HNB and LAMP-ELECTROPHORESIS methods in human and tick samples and their level of agreement.

	**LAMP- electrophoresis**			**kappa (k)**	**CI 95%**
	**Positive**	**Negative**	**Total**		
**HUMANS**					
**LAMP-HNB**					
Positive	50	0	50	0.82	0.63–1.0
Negative	10	43	53		
Total	60	43	103		
**TICKS**					
**LAMP-HNB**					
Positive	13	0	13	0.87	0.51–1.0
Negative	2	15	17		
Total	15	15	30		

95% CI, 95% confidence interval.

### 3.6 Diagnostics performance and likelihood ratio between PCR and LAMP methods

The diagnostics performance of the LAMP method when compared with the reference PCR method when human samples were used showed a sensitivity of 93% (95% CI: 77.9–99.2%) and a specificity of 70% (95% CI: 58.62–80.03%), in the human DNA samples when using the LAMP-HNB technique (Table 4). When the LAMP method was visualized by electrophoresis, the sensitivity obtained was 97% (95% CI: 82.7–99.9%) and the specificity was 58% (95% CI: 45.4–69%) (Table 4).

**Table 4:** Diagnostics performance of the LAMP-HNB and LAMP-ELECTROPHORESIS methods when compared with the reference PCR method in human and tick DNA samples.

	**HNB**	**IC 95%**	**Electrophoresis**	**IC 95%**
**Humans**				
Sensitivity	93%	77.9–99.2	97%	82.7–99.9
Specificity	70%	58.62–80.03	58%	45.4–69
+LR	3.1	2.16–4.45	2.28	1.73–3.0
–LR	0.1	0.02–0.37	0.06	0.01–0.4
**Ticks**				
Sensitivity	80%	51.9–95.67	87%	59.5–98.3
Specificity	93%	68–99.83	87%	59.5–98.3
+LR	12	1.78–81	6.52	1.76–24
–LR	0.2	0.08–0.59	0.15	0.04–0.57

LR+, positive likelihood ratio; LR–, negative likelihood ratio.

The positive likelihood ratio (+LR) obtained in LAMP-HNB was 3.1 (95% CI: 2.16–4.45) (Table 4). The negative likelihood ratio (–LR) was 0.1 (CI 95%: 0.02–0.37). For the LAMP method visualized by electrophoresis, the +RV was 2.28 (95% CI: 1.73–3.0), while the -RV was 0.06 (95% CI: 0.01–0.4). Therefore, according to Aznar-Oroval et al. (2013), the RV + of both visualization methods is considered low and the – RV is excellent.

On the other hand, the diagnostics performance of the LAMP method when compared with the reference PCR method when tick samples were used showed a sensitivity of 80% (95% CI: 51.9–96.67%) and a specificity of 93% (95% CI: 68–99.83%) using the LAMP-HNB technique. In the LAMP method visualized by electrophoresis, the sensitivity determined was 87% (95% CI: 59.5–98.3%) and a specificity of 87% (95% CI: 59.5–98.3%) (Table 4).

Additionally, the likelihood ratios obtained in LAMP-HNB, it was 12 (95% CI: 1.78–81) for LR + and LR– it was 0.2 (CI 95%: 0.08–0.59); in LAMP visualized by electrophoresis, LR + was 6.52 (95% CI: 1.76–24) and 0.2 (95% CI: 0.0–0.59) for LR–. Therefore, the LR + in LAMP-HNB is considered excellent (>10), and for the LAMP visualized by electrophoresis, it is considered good (5–10). The LR- of both is considered good (0.1–0.2) (Table 4). According to the results obtained, both methods could confirm or rule out suspected infection with the pathogen, with high confidence (Aznar-Oroval et al., 2013).

### 3.7 Positivity and negativity probability in LAMP

The positive and negative post-test probabilities, which is the probability that a patient is sick or not after applying the diagnostic test (van Stralen et al., 2009), were evaluated using the diagnostic test calculator (http://araw.mede.uic.edu/cgi-bin/testcalc.pl). As shown in Table 5, the result showed that in the LAMP-HNB technique, the positive post-test probability is 56% (95% CI: 46–64%), while the negative post-test probability is 4% (95% CI: 1–13%). These results indicate that 1 in 1.8 with a positive diagnosis are actually infected and 1 in 1 with a negative diagnosis are uninfected. For LAMP visualized by electrophoresis, the positive post-test probability is 48% (95% CI: 42–55%), while the negative post-test probability is 2% (95% CI: 0–14%) (Table 5). These results mean that 1 in 2.1 individuals with a positive diagnosis are infected and 1 in 1 individuals with a negative diagnosis are uninfected (Douglas, 1991; Schwartz, 2006).

**Table 5:** Pre-test and post-test probability analysis using LAMP.

	**HNB**	**CI 95%**	**Electroforesis**	**CI 95%**
**Humans**				
Prior odds	0.42		0.41	
Posterior odds (+)	1.3		0.9	
Posterior probability (+)	56%	46–64	48%	42–55
Posterior odds (–)	0.0		0.0	
Posterior probability (–)	4%	1–13	2%	0–14
**Ticks**				
Prior odds	1		1	
Posterior odds (+)	12		6.52	
Posterior probability (+)	92%	64–99	87%	64–96
Posterior odds (–)	0.2		0.1	
Posterior probability (–)	17%	7–37	13%	4–36

Additionally, the results obtained using tick samples for LAMP-HNB showed that the positive post-test probability was 92% (95% CI: 64–99%), and the negative post-test probability is 17% (95% CI: 7–37%) (Table 5). These results indicate that 1 in 1.1 ticks with a positive infection are infected and 1 in 1.2 ticks with a negative diagnosis are uninfected. On the other hand, for LAMP visualized by electrophoresis, the positive post-test probability is 87% (95% CI: 64–96%), while the negative post-test probability is 13% (95% CI: 4–36%). This results mean that 1 in 1.2 ticks with a positive diagnosis are actually infected, while 1 in 1.2 ticks with a negative diagnosis are uninfected (Douglas, 1991; Schwartz, 2006).

The sequence primers and the methodology were submited for patent as “SYNTHETIC OLIGONUCLEOTIDES SPECIFIC FOR THE DETECTION OF GENETIC MATERIAL OF *RICKETTSIA RICKETTSII* AND ITS DETECTION METHOD”, file number: MX/a/2022/003615. Date of submission: 20/04/2022.

## 4 Discussion

Globally, hundreds of RMSF cases are reported annually. This highly lethal tick-borne infectious disease is distributed throughout the Americas (Álvarez-López et al., 2021).^1^ However, as a result of delayed diagnosis or misdiagnosis, a substantial number of cases remain undetected, leading to significant morbidity and mortality. Therefore, diagnosis is essential for rapid and accurate detection of the pathogen (Yang and Rothman, 2004). Recent outbreaks of rickettsiosis underscore the critical need for the development of reliable and specific diagnostic methods, particularly those based on early and affordable detection, which ultimately help decrease mortality rates (Álvarez-Hernández et al., 2017; Jay and Armstrong, 2020). Diagnosis is critical for RMSF, as its symptoms are easily misattributed to other arthropod-borne diseases, such as dengue, Zika, and malaria, which require entirely different treatments.

Unfortunately, conventional methods for identifying *R. rickettsii* in hospitals or laboratories are associated with prolonged turnaround times and expensive tests. One of the major challenges in developing a good diagnostic method is that it must reduce the duration between the administration of the test and the availability of its results while being affordable and high in sensitivity to detect the pathogen, even in patients who have received antibiotic treatment.

The development of a novel diagnostic test should offer advantages compared to existing options, offering advantages such as reduced processing time, ease of access, and cost-effectiveness, and minimal invasiveness and risk (Trevethan, 2017). In this study, we have developed a simple, rapid (< 1 h), and cost-effective diagnostic approach. Considering the progression of the RMSF disease, a swift diagnosis can help the initiation of treatment, effectively managing the disease and thereby reducing mortality (Banoo, 2010).

The analytical sensitivity and specificity of LAMP assay for the detection of RMSF demonstrate that clinical samples can be analyzed without sophisticated equipment, as only an easy-to-use water bath is required. Therefore, this LAMP assay is a potentially valuable tool for the rapid detection of RMSF.

Generally, molecular detection methods such as PCR or qPCR require the use of specific primers for the *Rickettsia* genus; however, at the species levels, it is necessary to amplify and sequence different genes such as *gltA, ompA*, and/or *ompB* (Parola et al., 2005). Nevertheless, these genes are not divergent enough to distinguish between all rickettsial species (Roux and Raoult, 1993, 1995; Roux et al., 1996, 1997). Furthermore, the sensitivity was determined using 10-fold serial dilutions of plasmid DNA containing a partial sequence of an unknown gene. The sensitivity was found to be very high in the LAMP method (>10^−5^) compared to the PCR method. This could be due to the use of two specially designed loop primers. It has been shown, for other diseases, that the LAMP method exhibits a higher sensitivity when compared to conventional PCR, yet it is similar to real-time PCR (Nakao et al., 2013).

We evaluated and compared all the *Rickettsia* genomes in the available databases to obtain a specific sequence for the identification of *R. rickettsii* that was present in all available isolates. Many studies have shown variations in the pathogenic biotypes of *R. rickettsii* isolates and quantified their correlation with the clinical manifestations of RMSF (Karpathy et al., 2007; Eremeeva and Dasch, 2009). Specific primers were designed for sequences specific to *R. rickettsii*, with their specificity verified through bioinformatic analysis. Further *in vitro* verification was conducted using DNA samples from 18 *Rickettsia* species and other members of the *Rickettsia* genus, such as *A. phagocitophylum* and *E. chaffensis*, as well as other pathogens transmitted by ticks, such as *B. burgdorferi*.

Molecular detection methods can be expensive and demand specialized equipment and conditions, posing challenges for outdated or traditional hospital laboratories, especially in “fringe” or remote areas. The LAMP reaction can be visualized by detecting the DNA products in an agarose gel. However, this may not be necessary because a positive LAMP reaction causes the solution to become cloudy due to the formation of a magnesium pyrophosphate byproduct, and HNM is a colorimetric indicator that changes according to the concentration of Mg^2+^ ions depending on the pH of the solution (Goto et al., 2009). Some parameters (e.g., the turbidity of the solution) are highly correlated with the amount of DNA synthesized, and their measurement requires a real-time turbidimeter, which is prohibitively expensive. This cost factor not only reduces the versatility of LAMP but also significantly limits its potential widespread use of this procedure, especially in developing countries or “marginal” areas (Mori et al., 2001, 2004).

LAMP can be considered as an alternative to PCR-based methods for the early detection of *R. ricketssii* in scenarios where expensive or specialized equipment, such as thermocycler or real-time PCR, is not available; rather, it only requires the master mix, the DNA sample, and a heating block or water bath to maintain a constant temperature.

The results obtained using human clinical samples indicated a moderate agreement in precision and kappa index between HNB and electrophoresis, according to the evaluation criteria established by Landis and Koch (1977). Conversely, the results obtained using DNA from tick samples showed that the level of agreement was good for HNB and electrophoresis.

Additionally, the concordance between colorimetry and electrophoresis was estimated as visualization methods of the LAMP test results for both human and tick samples. These results indicate that there is a very good agreement between both visualization methods (Landis and Koch, 1977).

It should be noted that the discrepancy in the degree of concordance between the human and tick clinical samples may be due to the technique used. Specifically, the technique used for the human clinical samples was endpoint PCR, whereas for the tick samples, it was nested PCR, known for its higher sensitivity and specificity compared to endpoint PCR (Loeffelholz and Deng, 2006), for which there is a better degree of agreement with our LAMP method.

We observed that the results from HNB or electrophoresis did not align with the expected estimates (sensitivity 98%, specificity 95%, with a confidence of 95% and error of 5%). In the case of sensitivity, the difference is small, but the specificity is much lower than estimated. However, upon reassessment to achieve the expected values, it was determined that the number of samples should be increased for both indicators (OIE, 2018). The likelihood ratios, indicating the increased likelihood of obtaining a specific result (positive or negative), reveal that the LAMP method, when compared with the reference PCR method for human samples, exhibits a low RV+ for both visualization methods, while the RV– is excellent (Aznar-Oroval et al., 2013). In addition, when assessing the diagnostics performance of the LAMP method against the reference PCR method in tick samples, the LR+ in LAMP-HNB is considered excellent, while for the LAMP visualized by electrophoresis, it is considered good. The LR– of both methods is considered good. According to the results obtained, both methods could confirm and rule out suspected infection with the pathogen, with high confidence (Aznar-Oroval et al., 2013).

On the other hand, when assessing the positive and negative posttest probabilities, which is the probability that a patient is sick or not after applying the diagnostic test (van Stralen et al., 2009), the result indicated that the positive and negative posttest probabilities in the LAMP-HNB technique indicate that 1 in 1.8 with a positive diagnosis are infected and 1 in 1 with a negative diagnosis are uninfected. For LAMP visualized by electrophoresis, these results mean that 1 in 2.1 individuals with a positive diagnosis are infected and 1 in 1 individual with a negative diagnosis are uninfected (Douglas, 1991; Schwartz, 2006). Furthermore, the results obtained using tick samples for LAMP-HNB indicate that 1 in 1.1 ticks with a positive infection are infected and 1 in 1.2 ticks with a negative diagnosis are uninfected. On the other hand, for LAMP visualized by electrophoresis, these results mean that 1 in 1.2 ticks with a positive diagnosis are infected, while 1 in 1.2 ticks with a negative diagnosis are uninfected (Douglas, 1991; Schwartz, 2006). Therefore, when evaluating a diagnostic test, it is essential to understand and interpret its intrinsic properties (sensitivity and specificity). Recognizing that the predictive values and the degrees of probability are better indicators in practice, they play a crucial role in determining the usefulness of the method for diagnosis and aiding in decision-making (Trevethan, 2017).

In selecting a new diagnostic technique, it should not only possess intrinsic characteristics but also offer additional advantages compared to previous techniques, supporting its application. These advantages may include obtaining results in less time, a simpler application, a reduced cost, increased accessibility, minimal invasiveness and risk, user acceptance and, above all, the capability for technology transfer (Trevethan, 2017). Furthermore, the course of the disease should be taken into consideration, including whether it is severe, asymptomatic, or contagious. Therefore, a rapid diagnosis can be crucial in initiating timely treatment, especially when the course of the disease requires it, and it can be treated effectively, reducing the risk of it (Banoo, 2010). The LAMP test for *R. rickettsii* evaluated in this study offers comparative advantages with respect to the reference standards used. These advantages are the low cost of the test, prompt results, as well as the fact that its interpretation can be done using colorimetric visualization (HNB), or electrophoresis, since they present a high degree of agreement. LAMP is easier to perform compared to PCR, and above all, it does not require specialized equipment.

Currently, the application of LAMP for the molecular detection of *Rickettsia* spp., scrub typhus and murine typhus, has been reported (Dittrich et al., 2014; Hanaoka et al., 2017; Roy et al., 2021). According to the criteria of the World Health Organization (WHO, 2021), the new LAMP technique for *R. rickettsii* meets many of the requirements of a molecular detection method, including low cost, simplicity, speed, robustness, and easy availability of instruments and equipment.

## Data availability statement

The raw data supporting the conclusions of this article will be made available by the authors, without undue reservation.

## Ethics statement

The studies involving humans were approved by Bioethics Committee, Natural Sciences College, Autonomous University of Queretaro. The studies were conducted in accordance with the local legislation and institutional requirements. The human samples used in this study were acquired from gifted from another research group. Written informed consent for participation was not required from the participants or the participants' legal guardians/next of kin in accordance with the national legislation and institutional requirements. The animal study was approved by Bioethics Committee, Natural Sciences College, Autonmous University of Queretaro. The study was conducted in accordance with the local legislation and institutional requirements.

## Author contributions

BC-G: Formal analysis, Investigation, Methodology, Writing – original draft, Writing – review & editing. AO-B: Investigation, Validation, Writing – original draft, Writing – review & editing. LT-G: Resources, Writing – review & editing, Data curation, Supervision. GG-P: Investigation, Methodology, Resources, Supervision, Writing – review & editing. KD-R: Resources, Writing – review & editing, Validation. GA-T: Resources, Writing – review & editing, Data curation. MH-R: Data curation, Writing – review & editing, Investigation. JM: Writing – review & editing, Conceptualization, Funding acquisition, Resources, Supervision.

## Footnotes

^1^Centers for Disease Control and Prevention. *Rocky Mountain Spotted Fever, Epidemiology and Statistics*. Available online at: https://www.cdc.gov/rmsf/stats/index.html (accessed May 18, 2023).
